# Stigma Sensitivity and the Duration of Temporary Closure Are Affected by Pollinator Identity in *Mazus miquelii* (Phrymaceae), a Species with Bilobed Stigma

**DOI:** 10.3389/fpls.2017.00783

**Published:** 2017-05-10

**Authors:** Xiao-Fang Jin, Zhong-Ming Ye, Grace M. Amboka, Qing-Feng Wang, Chun-Feng Yang

**Affiliations:** ^1^Institute of Ecology and Environmental Science, Nanchang Institute of TechnologyNanchang, China; ^2^Key Laboratory of Aquatic Botany and Watershed Ecology, Wuhan Botanical Garden, Chinese Academy of SciencesWuhan, China; ^3^Jiangxi Provincial Key Laboratory of Soil Erosion and Prevention, Jiangxi Institute of Soil and Water ConservationNanchang, China; ^4^College of Life Sciences, University of Chinese Academy of SciencesBeijing, China

**Keywords:** *Mazus miquelii*, mechanical stimulation, pollen load, pollination efficiency, pollinator body size, pollinator selection, stigma behavior, touch-sensitive stigma

## Abstract

A sensitive bilobed stigma is thought to assure reproduction, avoid selfing and promote outcrossing. In addition, it may also play a role in pollinator selection since only pollinators with the appropriate body size can trigger this mechanism. However, no experimental study has investigated how the sensitive stigma responds to different pollinators and its potential effects on pollination. *Mazus miquelii* (Phrymaceae), a plant with a bilobed stigma was studied to investigate the relationship between stigma behaviors and its multiple insect pollinators. The reaction time of stigma closure after touched, duration of temporary closure, and factors determining permanent closure of the stigma were studied when flowers were exposed to different visitors and conducted with hand pollination. Manual stimulation was also used to detect the potential differences in stigmas when touched with different degrees of external forces. Results indicated that, compared to pollinators with a small body size, larger pollinators transferred more pollen grains to the stigma, causing a rapid stigma response and resulting in a higher percentage of permanent closures. Duration of temporary closure was negatively correlated with the speed of stigma closure; a stigma that closed more rapidly reopened more slowly. Manual stimulation showed that reaction time of stigma closure was likely a response to external mechanical forces. Hand pollination treatments revealed that the permanent closure of a stigma was determined by the size of stigmatic pollen load. For large pollinators, the speedy reaction of the stigma might help to reduce pollen loss, enhance pollen germination and avoid obstructing pollen export. Stigmas showed low sensitivity when touched by inferior pollinators, which may have increased the possibility of pollen deposition by subsequent visits. Therefore, the stigma behavior in *M. miquelii* is likely a mechanism of pollinator selection to maximize pollination success.

## Introduction

A sensitive stigma will close when touched by pollinator and may reopen after a visit. Behavior of a sensitive stigma reflects a special relationship between plants and animals by way of pollination (Darwin, 1876; Fetscher and Kohn, 1999; Fetscher et al., 2002; Sharma et al., 2008, and references therein). Plants with sensitive stigma are widely spread in the high core Lamiales (Newcombe, 1922, 1924; Angiosperm Phylogeny Group, 2009; Schaferhoff et al., 2010). Generally, behaviors of a sensitive stigma consist of three consecutive events, temporary closure, reopening and permanent closure (Newcombe, 1922, 1924). The stigma closes temporarily after being touched and reopens quickly or slowly after touching, and may close permanently when enough pollen has been deposited on the stigma (Newcombe, 1922, 1924; Bertin, 1982; Yang et al., 2004; Sritongchuay et al., 2010; Ai et al., 2013). The temporary closure of stigma may prevent self-pollination. The stigma receives pollen grains from a pollinator visiting from another flower and the visit may cause stigma closure until the pollinator flies away. Thus the pollen grains transferred by pollinators have a limited probability of being deposited on its own stigma (Newcombe, 1922; Sharma et al., 2008). In addition, a closed stigma may also enhance the efficiency of pollen removal (Fetscher et al., 2002). This purports a rapid reaction time of stigma temporary closure may enhance pollen export and effectively avoid self pollen deposition. Reopening appears to be a response to insufficient pollen deposited on a stigma for production of a full seed set (Fetscher, 1999). However, the correlation between the duration of temporary closure and pollen load size is still unclear. In addition, the closure status of a stigma has been considered effective for creation of the appropriate microenvironment for pollen to germinate and/or prevent the pollen from removal by subsequent pollinators or wind (Newcombe, 1922, 1924).

Behavior of sensitive stigma has been found to differ greatly between different plant taxa (Newcombe, 1922, 1924; Bertin, 1982; James and Knox, 1993; Gibbs and Bianchi, 1999; Yang et al., 2004; Hobbhahn et al., 2006; Qu et al., 2007; Guimaraes et al., 2008; Milet-Pinheiro et al., 2009; Rana, 2009; Sritongchuay et al., 2010; Jin et al., 2015) and within the same plants (Anderson, 1922; Sweety et al., 2009; Ren and Tang, 2010; Bittencourt et al., 2011; Ai et al., 2013). Since stigma sensitivity (reaction time of temporary closure after touched) and duration of temporary closure may be adaptive significance in pollination, studies on the effects of different pollinators on behaviors of a sensitive stigma would be of importance in understanding the evolution of sensitive stigmas. However, whether stigma sensitivity, the duration of temporary closure, and permanent closure are mediated by similar or different factors still remain unclear. In addition, the behavior of a bilobed sensitive stigma should be regarded as a mechanism of pollinator selection because only pollinators with the appropriate body size can trigger stigma closure. However, to our knowledge, no experimental studies have been conducted to investigate the effects of different pollinators on behavior of a sensitive stigma, especially for plant species with a variety of pollinators.

**FIGURE 1:**
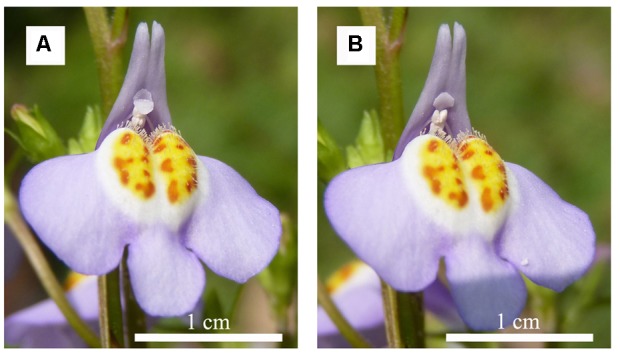
**Flower morphology and size of *Mazus miquelii*; (A)** indicating a flower with an open stigma and **(B)** indicating a flower with a closed stigma.

We hypothesized that behavior of a bilobed sensitive stigma may vary in response to a variety of pollinators with different body sizes, which could be mediated by variations in pollination efficiencies. In this study, *Mazus miquelii* Makino, a plant with a bilobed stigma (**Figure 1**) was used to test this hypothesis. *M. miquelii* is visited by several pollinators with different body sizes including Anthophorine bees, *Osmia* spp., *Lasioglossum* spp., and *Halictus* spp. (Jin et al., 2015). We addressed the following questions: (a) Do pollinators with different body sizes cause differences in stigma behavior? Reaction time of temporary closure after touched, duration of temporary closure, and the probability of permanent closure were studied when enclosed flowers were exposed to different pollinators. (b) Are there any differences in factors determining reaction time of temporary closure after touched, duration of temporary closure, and permanent closure of the stigma?

## Materials and Methods

### Study Species and Site

*Mazus miquelii* Makino (Phrymaceae) is a perennial herb that grows in wet places by trails and sparse forests. It is widespread in central and eastern China and is also found in Japan and North eastern America as an introduced species. *M. miquelii* can reproduce both sexually with seeds and asexually with a short stolon. Usually, each plant produces one to 16 inflorescences each with 13–20 whitish to blue flowers. One to 10 inflorescences are available at the same time in a given individual. The flowers often open at about 9 AM and each flower stays open for 2–5 days (Jin, 2015). A flower consists of upper and lower lips with four anthers and style incorporated in the upper lip. In addition, the flower produces nectar at the bottom of corolla tube. The bilobed stigma is located adjacent to the corolla-opening and sheltered by the upper lip, which is the route for pollinators foraging for pollen or nectar (Jin et al., 2015). Furthermore, an inflorescence yields one flower in a day, each flower producing about 400 ovules (Jin et al., 2015). *M. miquelii* is partially self-compatible, nonetheless it is incapable of setting seeds without pollination (Kimata, 1978; Jin et al., 2015).

Field investigation was conducted in 2014 on a naturally occurring population with at least 500 individual plants at the Wuhan Botanical Garden (WBG), Hubei Province, China. Leaf-cutter bees (Megachilidae: *Osmia rufina* and *Osima jacoti*), Anthophorine bees (Anthophoridae: *Habropoda tainanicola*, *Tetralonia chinensis*, and *Tetralonia jacoti*), and sweat bees [Halictidae: *Halictus (Seladonia) aerarius*, *Halictus (Seladonia) varentzowi*, and *Lasioglossum* sp.] were the primary pollinators of *M. miquelii* at the study site during the study period (Jin et al., 2015). In this study, pollinators were divided into four groups according to body size, namely *Halictus* spp., *Lasioglossum* spp., Anthophorine bees, and *Osmia* spp. Body sizes and foraging behaviors of individuals among species within the same group were similar (Jin et al., 2015). About 100 plants were randomly selected and enclosed with fine mesh netting for pollinator exclusion before the flowers opened. The plants were either exposed to pollinators or artificial manipulation when the flowers opened.

### Pollination Observations and Stigma Behavior in Response to Different Pollinators

Field investigations were conducted from 11 am to 5 pm on April 1–9, 2014. Foraging behaviors of each group of the pollinators were carefully observed in sunny days. The floral resource sought by each pollinator group was recorded when the pollinator came into contact with the stigma and/or anther. Pollinator found grooming pollen from anthers was described as pollen foragers whilst those that licked the nectar at the flower bottom were termed as nectar foragers. The body size of each pollinator group was measured by a vernier caliper. Ten individuals from each of the pollinator groups were used for measurement of body size. The *Halictus* spp. and *Lasioglossum* spp. entered into the floral tube while the latter stayed at the flower opening to seek for nectar (Jin, 2015). We then measured the body diameter of *Halictus* spp. and *Lasioglossum* spp., and the head diameter of Anthophorine bees and *Osmia* spp. to estimate the body size of the pollinators.

Different pollinators may have different abilities of depositing pollen on a stigma. The enclosed flowers were used to evaluate this variance. Flowers that received a single visit by a pollinator were enclosed immediately and picked on the day after being visited and fixed in FAA solution (formalin: acetic acid: 70% ethanol at a ratio of 5:5:90 by volume) for further analysis in the laboratory. Stigmatic pollen load was counted under a fluorescence microscope (Nikon E-600) after treatment with 8 mol/L NaOH for 10 h followed by 0.1% aniline blue dye. The sampled flowers were 27, 18, 27, and 36 for *Halictus* spp., *Lasioglossum* spp., Anthophorine bees, and *Osmia* spp., respectively. The duration of a single visit for each group of the pollinators was recorded, from landing on the flower to taking off. In this survey, we recorded 21, 22, 18, and 23 visits for *Halictus* spp., *Lasioglossum* spp., Anthophorine bees, and *Osmia* spp., respectively.

To study stigma behavior in response to the pollinators with different body sizes, enclosed plants were exposed to pollinators of each group separately when the flowers were fully open. A flower that received a single visit from a pollinator that had previously visited another *M*. *miquelii* flower was marked with a string with different color for marking pollinators from different groups. The plant was again enclosed after the flower received a visit for observation of the stigma behavior. The stigma behavior was recorded in three aspects: percentage of temporary closure, duration of temporary closure (min) and percentage of permanent closure. The reaction time of stigma temporary closure during pollination observations was not measured due to difficulties in estimating the exact time of contact between the pollinator and the stigma. However, this was manipulated by artificial stimulation. To detect the differences in the percentage of temporary closure after a single visit among pollinator groups, it was observed that 27, 20, 10, and 30 flowers received a single visit by *Halictus* spp., *Lasioglossum* spp., Anthophorine bees, and *Osmia* spp., respectively. The stigmas closed after visitation but reopened later were recorded as a temporary closure. The duration of temporary closure (min) when flowers received a single visit by different pollinators was recorded as 8, 20, 10, and 30 flowers were visited by *Halictus* spp., *Lasioglossum* spp., Anthophorine bees, and *Osmia* spp., respectively. The duration of temporary closure indicated the period from the moment of stigma temporary closure to that of reopening. The percentage of permanent closure when flowers received a single visit by different pollinators was recorded as 27, 20, 10, and 30 flowers visited by *Halictus* spp., *Lasioglossum* spp., Anthophorine bees and *Osmia* spp., respectively. Permanent closure was observed for the stigma of enclosed flower (after a single visit) as it closed automatically and did not reopen until flower wilt. The interval between flower visited by a pollinator and stigma permanent closure was recorded.

One-way ANOVA was used to detect the differences of different pollinators on duration of stigma temporary closure. The foraging duration and body size of pollinators from different pollinators were also analyzed by a one-way ANOVA test. A *post hoc* test was used for multiple comparisons when a significant difference was observed. Chi-squared test was used to examine the differences in the influences of different pollinators on percentage of temporary and permanent closure. Fisher’s exact test was used and the frequency was weighted before the Chi-squared test. Partitions of the χ^2^ method were used in pairwise comparison.

### Stigma Behavior in Response to Manual Stimulation and Hand Pollination

To disentangle the behavior of the stigma in response to mechanical touch, manual stimulation was applied to the flower. We used three sizes of cotton balls, namely 2, 3.5, and 4.5 mm in diameter, to create different external forces when touching the stigma. Former studies (Jin, 2015) revealed that the height of corolla-opening (the distance from lower stigma lobe to the lower corolla lip) was about 2.5 mm for *M*. *miquelii* flowers with an open stigma. This distance was similar to the smaller cotton ball but shorter than the two bigger cotton balls. When the cotton balls with different sizes were pushed through the corolla-opening, they should create different levels of external force on stigma. We then named the forces evoked by the cotton balls as small, medium and large forces, respectively.

Stimulations of each level of forces on stigma were replicated on at least 30 newly opened flowers from previously enclosed plants. The reaction time of stigma temporary closure was recorded (in seconds, from the moment of interaction to the moment of complete closure of the two lobes) and duration of stigma temporary closure (in minutes, from stigma temporary closure to reopening of the two lobes). The relationship between reaction time of temporary closure and duration of stigma temporary closure was tested. Likewise, we included flowers (*N* = 10) in their 2nd day of opening in the manual stimulation experiment to detect whether stigma sensitivity was influenced by flowering stage. Furthermore, to determine whether the stigma repeatedly closed and reopened during anthesis of a flower, mechanical touch with a large force was applied to the stigma as soon as it opened or reopened. One-way ANOVA was used to detect the effects of different external forces on reaction time of stigma temporary closure and duration of stigma temporary closure and followed by a *post hoc* test for multiple comparisons when a significant difference was found. Unary Linear Regression Model was used to analyze the relationship between stigma reaction time of temporary closure and duration of temporary closure.

The influence of different pollen sources on stigma behavior was detected in *M. miquelii*. Pollen from the same plant, pollen from another individual (1 m in distance), pollen from another *Mazus* species (*M*. *pumilus*), and pollen from a co-flowering plant in the habitat (*Glechoma longituba*) were used as the sources of pollen. For each pollen source, hand pollination with large cotton ball was conducted on 10 flowers each from different individuals. Moreover, stigma of each of the flowers was deposited on more than 1,000 pollen grains (more than twice of the ovule number per flower). Before hand pollination, pollen grains were deposited on a glass slide and counted under a portable microscope. For each of the treatments, we recorded the reaction time of stigma temporary closure and duration of stigma temporary closure. The results were also compared with those of the manual touch with a large force. One-way ANOVA was used in analysis of different pollen sources on reaction time of stigma temporary and duration of stigma temporary closure. A *post hoc* test was used for multiple comparisons when a significant difference was detected by one-way ANOVA.

To detect whether stigma permanent closure was determined by pollen load size, various amounts of pollen grains from other individuals were deposited on stigma of different flowers (*N* = 70). All flowers used for hand pollination were from enclosed plants. On the 2nd day, flowers with open and closed stigma were fixed in FAA solution for subsequent counting of pollen load size. Load size was later counted under a fluorescence microscope (Nikon E-600) after treatment with 8 mol/L NaOH for 10 h followed by dyeing with 0.1% aniline blue. We recorded the pollen load size of each stigma with open or closed status and moreover, we calculated the percentage of stigmas with permanent closure out of total stigmas checked under different pollen load sizes. All the data was analyzed with SPSS 18.0.

## Results

### Stigma Behavior in Response to Different Pollinators

Among the flower visitors, Anthophorine bees and *Osmia* spp. foraged for nectar while *Lasioglossum* spp. and *Halictus* spp. collected both pollen and nectar. In addition, they differed significantly in body sizes. Individuals of *Halictus* spp. were the smallest while those of Anthophorine bees had the largest body sizes (*F*_3,36_ = 240.002, *P* < 0.001, **Table 1**). When newly opened flowers visited, respectively, by pollinators from the four groups, the percentages of flowers with a closed stigma out of total flowers differed significantly (χ^2^ = 49.815, *P* < 0.001). All stigmas from flowers visited by Anthophorine bees, *Lasioglossum* spp., and *Osmia* spp. closed, whereas only 29.6% of the stigmas from flowers visited by *Halictus* spp. closed (**Table 1**). However, all closed stigmas reopened while the duration of stigma temporary closure among flowers visited by different pollinators was significantly different (*F*_3,64_ = 13.966, *P* < 0.001). Stigmas of flowers visited by *Halictus* spp. had a shorter duration of temporary closure than those visited by pollinators from the other three groups (**Table 1**).

**Table 1:** Stigma behavior when flowers were exposed to different insects, and foraging behavior and efficiency for each pollinator.

Flower visitors	*Halictus* spp.	*Lasioglossum* spp.	Anthophorine bees	*Osmia* spp.
**Stigma behavior in response to different pollinators**				
Percentage of temporary closure after a single visit	29.6% (*n* = 27)^a^	100% (*n* = 20)^b^	100% (*n* = 10)^b^	100% (*n* = 30)^b^
Duration of temporary closure (min)	5.11 ± 0.58 (*n* = 8)^a^	7.19 ± 1.00 (*n* = 20)^b^	8.46 ± 2.13 (*n* = 10)^bc^	8.49 ± 1.49 (*n* = 30)^c^
Percentage of permanent closure after a single visit	7.4% (*n* = 27)^a^	85.00% (*n* = 20)^b^	60.00% (*n* = 10)^b^	83.33% (*n* = 30)^b^
**Behavior and efficiency of different pollinators**				
Foraged resource	Pollen, nectar	Pollen, nectar	Nectar	Nectar
Contact with sexual organs (s)	Anther, sometimes with stigma	Anther, often with stigma	Anther and stigma	Anther and stigma
Pollen deposition after single visit	42.11 ± 11.71 (*n* = 27)^a^	662.00 ± 87.33 (*n* = 18)^b^	572.37 ± 84.16 (*n* = 27)^b^	398.14 ± 68.68 (*n* = 36)^b^
Visit duration per flower (s)	30.37 ± 2.36 (*n* = 21)^a^	18.37 ± 1.59 (*n* = 22)^b^	1.85 ± 0.06 (*n* = 18)^d^	3.65 ± 0.17 (*n* = 23)^c^
Body/head diameter (mm)	1.64 ± 0.03 (*n* = 10)^a^	2.79 ± 0.10 (*n* = 10)^b^	4.34 ± 0.20 (*n* = 10)^d^	3.56 ± 0.25 (*n* = 10)^c^


*Data are mean ± SE with sample size in parentheses. Values with the same super scripted letters are not significantly different (P < 0.05)*.

For flowers with permanently closed stigmas, the time from flower visited by a pollinator to stigma permanent closure was about 8 h with no observable differences between open and enclosed flowers. That is, before permanent closure, the stigma repeatedly closed and reopened in response to touch by pollinators with large body size, e.g., Anthophorine bees, *Lasioglossum* spp., and *Osmia* spp. For the enclosed flowers which received a single visit by different pollinators, stigmas of some flowers closed automatically and stayed a status of closure after 8 h from the time of flower visited by a pollinator. The percentage of stigma permanent closure was significantly different among flowers visited by pollinators from the four groups (χ^2^ = 44.713, *P* < 0.001); as it was lower in *Halictus* spp. than in the pollinators from other three groups (**Table 1**).

Results indicated that pollen load on a stigma after a single visit differed significantly among flowers visited by different pollinators (*F*_3,101_ = 14.086, *P* < 0.001). *Halictus* spp. deposited significantly less pollen on the stigma than pollinators from the other three groups, and there was no significant difference among them (**Table 1**). Duration of flower foraging time also differed significantly among pollinators from the four groups (*F*_3,80_ = 84.435, *P* < 0.001); Anthophorine bees had the shortest foraging duration while *Halictus* spp. had the longest duration (**Table 1**).

### Stigma Behavior in Response to Manual Stimulation and Hand Pollination

**FIGURE 2:**
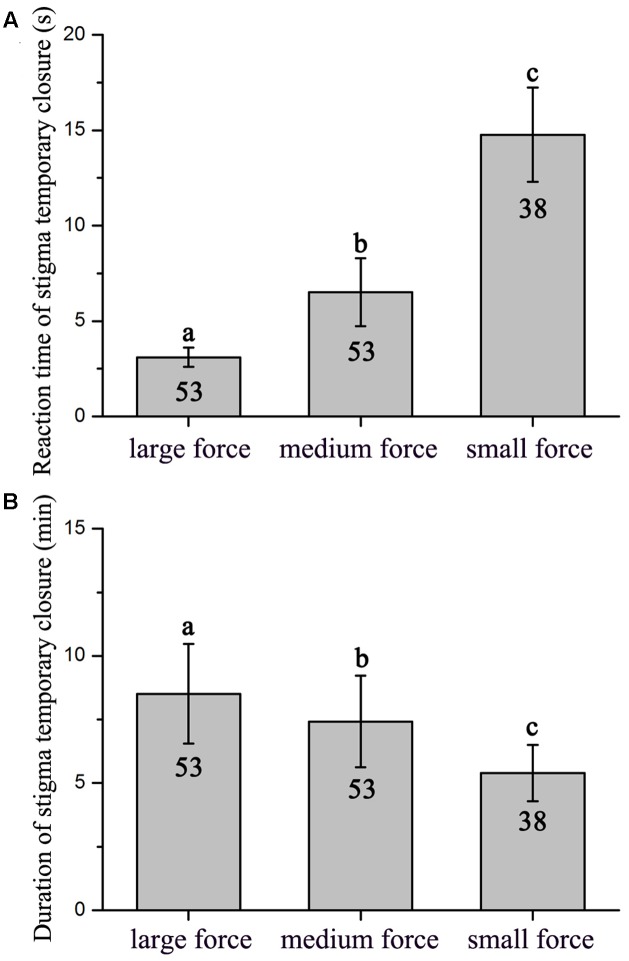
**Reaction time of stigma temporary closure (A)** and duration of stigma temporary closure **(B)** under different levels of mechanical forces (Mean ± 1 SD). Values with the same super scripted letters are not significantly different (*P* < 0.05).

**FIGURE 3:**
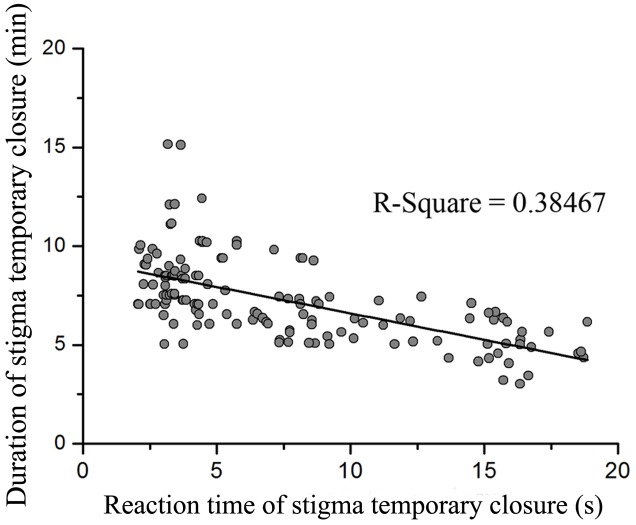
**The relationship between reaction time of stigma temporary closure and duration of stigma temporary closure when mechanically stimulated with different forces (*P* < 0.001)**.

Flowers did not display a significant difference between the 1st and 2nd day on reaction time of stigma temporary closure (*F*_1,18_ = 0.053, *P* = 0.820) and duration of stigma temporary closure in flowers (*F*_1,18_ = 0.640, *P* = 0.434). The stigma closed and reopened repeatedly in response to manual stimulation within the whole flowering period. However, reaction time of stigma temporary closure and duration of temporary closure was significantly different in response to touch with small, medium, and large forces (reaction time of stigma temporary closure: *F*_2,141_ = 155.094, *P* < 0.001, **Figure 2A**; duration of temporary closure: *F*_2,141_ = 29.704, *P* < 0.001, **Figure 2B**). Moreover, a significant negative relationship between the reaction time of stigma temporary closure and duration of temporary closure was identified [*Y* = 9.267 (±0.248)–0.267 (±0.028) X, *R*^2^ = 0.38, *df* = 143, *P* < 0.001, **Figure 3**]. The stigma of the flowers under manual stimulation did not close permanently despite the touch from small, medium, and large forces.

Different pollen sources and mechanical touch with a large force showed no significant differences in reaction time of stigma temporary closure (*F*_4,45_ = 0.855, *P* = 0.498) and duration of temporary closure (*F*_4,45_ = 0.447, *P* = 0.774). Hand pollination disclosed that stigma permanent closure was highly dependent on the size of stigmatic pollen load. After 8 h of hand pollination, it was observed that stigmas remained open when pollen load was lower than 400 pollen grains; 44% of stigmas remained open when pollen load was between 400 and 600 pollen grains and all the stigmas closed permanently when pollen load was higher than 600 pollen grains.

## Discussion

Stigma behavior of *M. miquelii* differed significantly when foraged by pollinators with different body sizes. Pollinators with a smaller body size, for example, *Halictus* spp., triggered a slower response in stigma closure but a shorter duration of stigma temporary closure. It could be attributed to the fact that small pollinators made limited contact with stigmas and thus imposing a small mechanical force on the stigma. This argument was supported by results of manual stimulation that small external forces caused a slower reaction time of stigma closure and a shorter duration of stigma temporary closure. Hand pollination revealed that stigma permanent closure was determined by load size of legitimate pollen grains (see also Yang et al., 2004; Sritongchuay et al., 2010; Jin et al., 2015; but see Milet-Pinheiro et al., 2009). The results indicated that it may be different in factors influencing reaction time of stigma closure, duration of stigma temporary closure, and stigma permanent closure.

Manual stimulations suggested that in *M. miquelii*, reaction time of stigma temporary closure was likely a response to the mechanical forces imposed upon the stigma. A bigger force resulted in a more rapid reaction of stigma temporary closure. Although the duration of stigma temporary closure was correlating to pollen source in a self-incompatible plant, *Anemopaegma chamberlaynii*, the closed stigma did not reopen after treatment of outcross-pollination but did reopen under self-pollination treatment (Correia et al., 2006), our results of hand pollination treatments indicated that in *M. miquelii*, neither reaction time of stigma temporary closure nor duration of stigma temporary closure was affected by different pollen sources. This was also observed in *Oroxylum indicum* (Bignoniaceae) (Sritongchuay et al., 2010). The duration of stigma temporary closure was negatively related to the reaction time of stigma temporary closure. Stigma temporary closure was likely an instant reaction to mechanical stimulation by external forces. Larger forces stimulated a wider stigma surface than smaller forces thus triggering a rapid response and complete closure of the stigma; therefore, the stigma would need a longer time to recover.

In *M. miquelii*, the novel finding that reaction time of stigma temporary closure was negatively correlated with duration of stigma temporary closure might contribute to facilitate pollination. Compared to larger pollinators, those with a smaller body size had lower pollen transfer efficiency resulting to a longer reaction time of stigma temporary closure but a shorter duration of stigma temporary closure. The slow closure and rapid reopening of a stigma increased the possibility of pollen deposition for the flower in subsequent visits. For the ongoing visit, the reopening stigma may be weak in obstructing pollen removal for a small pollinator. On the other hand, pollinators with a large body size deposited a large amount of pollen grains on a stigma and resulted in rapid stigma temporary closure; and the stigma maintained the status of temporary closure for a long time. A closed stigma might not only reduce pollen loss (Newcombe, 1922) and provide a comfortable microenvironment for pollen germination (Newcombe, 1922; Edlund et al., 2004), but also enhance the efficiency of pollen removal (Fetscher et al., 2002). In addition, a higher probability of stigma permanent closure caused by larger pollinators could facilitate pollination since larger pollinators may deposit more pollen grains on the stigma compared to pollinators with a smaller body size (Richardson, 2004; Yang et al., 2004; Milet-Pinheiro et al., 2009). Besides, larger pollinators normally have a greater flight capability; thus capable in transporting pollen from very distant plants of different populations and in turn benefiting plants by increasing rate of outcrossing and assuring a constant gene flow (Schlindwein et al., 2014). The sensitive stigma in *M. miquelii* might be a mechanism of pollinator selection that maximize plant reproductive success.

Stigma permanent closure was deemed to be an indication of enough pollen grains deposited on a stigma to fulfill full seed set (Yang et al., 2004; Sritongchuay et al., 2010; Jin et al., 2015). This study supported the argument because permanent closure of *M. miquelii* stigmas required a minimum pollen load on stigma. Otherwise, the stigma remained opened for receiving pollen. Sufficient pollen grain deposition is essential to maximize female reproductive output (Fetscher, 1999). Consequently, when pollen load meet the demand of seed yield, stigma temporary closure could benefit male fitness by avoiding obstruction for pollen removal (Fetscher et al., 2002). Permanent stigma closure in response to pollen load size was therefore suggested to be a mechanism facilitating pollination (Yang et al., 2004). The previous study suggested that stigma permanent closure in *Mazus* might be facilitated by adequate growth pollen tubes in the style (Jin et al., 2015). However, direct experimental evidence is needed to support this hypothesis.

## Conclusion

This study revealed the relationship between stigma behavior and pollinators with different body sizes for a plant species with a sensitive bilobed stigma, *M. miquelii*. A larger pollinator deposited more pollen grains on stigmas resulting in higher possibility of stigma permanent closure than a small pollinator. Compared to larger pollinators, stigmas touched by pollinators with a smaller body size had a longer reaction time of stigma temporary closure, maintained the status of temporary closure for a shorter time and thus, had a relatively longer time to receive pollen grains. The stigma behavior in *M. miquelii* might be regarded as a mechanism of pollinator selection to maximize pollination success. However, further studies should focus on the physiological basis of stigma behavior to uncover the internal factors controlling reversible stigma opening (closure and reopening) and the completion of this process (permanent closure); other studies on touch-sensitive plant might be essential for referencing (Cameron et al., 2002; Braam, 2005; Forterre, 2013; Kruse et al., 2014).

## Author Contributions

X-FJ, Q-FW, and C-FY designed the study. X-FJ and Z-MY carried out the fieldwork. X-FJ, Z-MY, GA, and C-FY analyzed the results and wrote the manuscript.

## Conflict of Interest Statement

The authors declare that the research was conducted in the absence of any commercial or financial relationships that could be construed as a potential conflict of interest.
